# Antibiotic-Impregnated Bone Grafts in Orthopaedic and Trauma Surgery: A Systematic Review of the Literature

**DOI:** 10.1155/2012/538061

**Published:** 2012-07-26

**Authors:** Konstantinos Anagnostakos, Katrin Schröder

**Affiliations:** Klinik für Orthopädie und Orthopädische Chirurgie, Universitätskliniken des Saarlandes, Kirrbergerstraße 1D, Saar, 66421 Homburg, Germany

## Abstract

There exist several options for local antibiotic therapy in orthopaedic and trauma surgery. Over the past years, the use of antibiotic-impregnated bone grafts (AIBGs) has become a popular procedure in the treatment of bone and joint infections. A major advantage of AIBGs involves the possibility of impregnation of various antibiotics depending on the sensitivity profile of the causative organism, whereas an additional surgery with removal of the antibiotic carrier is not necessary, as in the use of antibiotic-loaded bone cement. However, generalized conclusions cannot be clearly drawn from the existing literature due to differences of bone used, impregnation method, antibiotics, their doses, laboratory circumstances, or clinical indications. The present work reviews the literature regarding this topic and sheds some light onto the choice of bone and antibiotics, manufacturing details, and clinical experience.

## 1. Introduction

Despite numerous advances in prophylactic measures, infections still remain a major complication in orthopaedic and trauma surgery. Depending on the infection localization, time of infection manifestation, presence of hardware, pathogen organism, its virulence, and antibiotic sensitivity profile, several treatment options are available, mostly consisting of surgical revision, systemic, and local antibiotic therapy.

There exist numerous devices for local antibiotic therapy. Ideally, all these devices should release high-antibiotic concentrations over a prolonged time period in order to not only eradicate the infection but also prevent a recurrent infection caused by bacteria that might have survived after the antibiotic concentrations have fallen to subinhibitory levels. Generally, these media can be divided into biodegradable ones (e.g., collagen sponges) and those which have to be removed in a further surgery (e.g., cement spacers or beads). Collagen sponges elute sufficiently antibiotics over 7–14 days in vivo [1]. Cement device may release antibiotics up to 30 days after implantation, whereas the majority of the eluted antibiotic amounts occur within the first 48 hours [2]. However, in the past years an increased number of studies have indicated that bacteria are capable of adhering to or even colonize antibiotic-loaded cement so that concerns have been raised with regard to prolonged implantation periods and the possibility of recurrent or persistent infections [3]. A possible solution might be the use of biodegradable device with superior pharmacokinetic properties than those of collagen sponges, but also with the option of an additional impregnation of antibiotic(s) depending on the particular causative organism. 

A carrier that might solve this problem is bone itself. The concept of impregnating bone with antibiotics is not a new idea. De Grood was the first to report on mixing penicillin with cancellous bone when filling bone defects in 1947 [4]. Two patients were successfully treated for residual cavities due to osteomyelitis. Although this idea seemed promising, it was not until the mid 80's that further reports have been presented at different scientific meetings [5–7]. Since then, various reports have been published about the use of antibiotic-impregnated bone grafts in in vitro, animal, and in vivo studies (Table 1). 

Despite the increasing popularity of this treatment option, generalized conclusions still cannot be drawn from the existing literature due to differences of bone and antibiotics used, impregnation method and dose, laboratory circumstances, or clinical indications. Hence, the aim of the present work was to review the literature regarding this topic and shed some light onto the choice of bone and antibiotics, manufacturing details, and clinical experience. 

## 2. Inclusion of Studies

A systematic literature search was carried out through Medline until 2010. Search terms included “bone (allo)graft(s),” and “antibiotic-loaded/impregnated,” alone and in combination. Only articles about orthopaedic and trauma surgery were evaluated except for those deriving from other surgical facilities but providing new information to this topic. From the initially retrieved studies, a further search was made throughout the bibliography of the identified publications for inclusion of as many studies as possible. Only English publications were included into the review process. Reviews and case reports were excluded from the study unless they provided new information which was not reported in the other identified publications. A total of 35 studies could be identified [7–41].

## 3. Bone Graft Choice

Based on the experience gained from aseptic surgery, the great majority of the studies report on the use of cancellous bone grafts (Table 1). Due to the known osteoconductive properties, cancellous bone carries a lower, theoretical risk of sequester formation compared with cortical bone which is of great importance in the treatment of bone infections. 

Depending on the surgical indication itself, infection localization, size of bone defect, and presence of an internal bone bank, either autologous or allogenous bone graft might be used. Generally, there are three types of bone allograft available: (i) fresh or fresh-frozen, (ii) freeze-dried, and (iii) demineralised freeze-dried. Despite the wide use of all bone graft types, to the best of our knowledge, there exists no information whether there is a difference regarding the pharmacokinetic properties among the groups. Moreover, it is unclear whether the manual fragmentation of the graft before or after the impregnation process might also have a theoretical impact on the pharmacokinetic properties of AIBGs.

## 4. Antibiotic Loading: Drug Choice and Impregnation Methods

Knowledge about the pharmacokinetic properties of bone itself but also about the possible influence of the incorporated antibiotics on the physical properties of the bone is an essential premise before clinical use of antibiotic-loaded bone grafts. Theoretically, all antibiotics might be appropriate for bone impregnation as long as they are eluted to sufficient amounts. In contrast to acrylic bone cement, where the polymerization heat might lead to an inactivation of the incorporated agents [2], the physical properties of the antibiotics are not influenced by the impregnation process at the site of AIBGs.

Although no specific guidelines exist, the drug choice should be generally made in accordance with the sensitivity profile of the causative pathogen organism (if preoperatively known). Otherwise, a calculated local antibiotic therapy should be applied, which should be effective against frequent pathogen organisms in orthopaedic surgery (e.g., *Staphylococci*). Literature data report mostly on monoantibiotic-loading. More detailed information about this topic is discussed later in the paper.

Regarding the impregnation process, several methods are described in the literature (Table 1). Some authors incorporate the antibiotics by manual mixing, whereas others place the bone grafts into antibiotic-containing solutions for various time periods. In some cases, the harvested bone graft has been specially prepared before antibiotic impregnation [34]. Which of the methods leads to the highest antibiotic impregnation remains, however, unknown.

Beside the aforementioned methods, single reports have indicated that iontophoresis might also be a novel method for incorporating antibiotics into bone [17, 24, 27]. Under experimental conditions, high levels of gentamicin and flucloxacillin could be initially released, whereas sufficient amounts could be eluted for a period of up to two weeks and the antibiotics have remained biologically active [17]. 

Furthermore, there exist great discrepancies regarding the exact antibiotic/bone graft ratio. Some authors define the impregnation amount per femoral head or grams of bone, whereas others determine this according to the bone volume (Table 1). This makes a comparison of the literature extremely difficult. Intraoperatively, harvested bone may greatly vary with regard to weight or cannot be compared with a femoral head depending on the harvesting area (e.g., anterior iliac crest) due to differences regarding the cancellous structure or the bone density. Therefore, for future studies it might be advisable to define the antibiotic amounts in accordance to the volume of bone used which is easier to determine intraoperatively than the weight.

## 5. Pharmacokinetic Properties

Knowledge about the elution kinetics of AIBG's is an indispensable premise for the successful planning and treatment of bone and joint infections. Numerous publications provide information about this topic, mostly consisting of in vitro and animal studies.

For other local antibiotic-impregnated carriers, such as bone cement, gentamicin is regarded to be the antibiotic with the best pharmacokinetic properties [2]. However, in case of AIBG's, other antibiotics have been proven to be superior. In an in vitro study, Winkler and colleagues could demonstrate that vancomycin is significantly better eluted from cancellous bone in comparison with tobramycin [34]. Witsø et al. investigated the release kinetics of 8 antibiotics from cancellous bone and reported an elution for up to 21 days with rifampicin having the longest and betalactamase the shortest one [37]. Kanellakopoulou et al. compared the elution of fusidic acid and teicoplanin from cancellous bone in vitro [20]. While allografts impregnated with fusidic acid could release high concentrations for the first 20 days, those loaded with teicoplanin showed low concentrations after the first 4 days.

The antibiotic concentrations eluted from antibiotic-impregnated bone grafts vastly exceed those from other local antibiotic-loaded carriers [11, 21, 34, 36]. Kanellakopoulou et al. determined highest moxifloxacin concentrations exceeding 4,500 *μ*g/mL on the first day under experimental conditions [21]. Winkler et al. compared in vitro the elution kinetics of cortical and cancellous bone impregnated with vancomycin or tobramycin [34]. Highest initial vancomycin concentrations were meanly 20,900 *μ*g/mL and 5,700 *μ*g/mL, respectively (the difference was significant). The release of tobramycin was significantly lower than that of vancomycin. Buttaro et al. reported highest vancomycin concentrations of 1,400 *μ*g/mL from bone grafts used in the treatment of infected hip arthroplasties [11]. For the same surgical indication, Winkler et al. could measure mean vancomycin levels in the drainage fluid of 535 *μ*g/mL on the first postoperative day, declining to 400 *μ*g/mL on the third day [36]. Although high local concentrations are eligible for a successful eradication of the infection, it should be born mind that these concentrations might be associated with an accompanying toxic effect on cells. High concentrations of vancomycin have been reported to substantially reduce osteoblast replication and even cause cell death [42, 43].

In case of a biantibiotic combination, a synergistic effect has been described between aminoglycosides and glycopeptides when eluted from acrylic bone cement [2]. This effect might appear for a single or both antibiotic groups depending on the antibiotic ratio [2]. To our knowledge, there exists only a single study which tried to investigate this effect at the site of AIBGs. Witsø et al. reported that the amount of vancomycin eluted from vancomycin-netilmicin-loaded grafts was significantly reduced compared with those loaded only with vancomycin, whereas the release of netilmicin was similar in both groups [39]. Whether this effect might vary depending on the antibiotic ratio of these agents or also counts for other antibiotic groups remains unclear. 

Rhyu et al. investigated whether a demineralization process would have a positive impact on the pharmacokinetic properties of vancomycin-loaded cancellous bone and whether the addition of blood would act as a biological barrier to control the drug release rate [32]. The authors could demonstrate a significant difference regarding the amount of the antibiotic uptake between demineralized and undemineralized bone for the former. Moreover, the addition of blood significantly slowed down the antibiotic elution over the first 7 days in vitro.

## 6. Histopathological, Radiographical, and Immunohistochemical Findings in Animal Studies

During a bone-grafting operation, there is usually a delay between procurement of the graft and its implantation. The delay might range from a few minutes to several hours. This raises several practical questions concerning the harmful effects of delay on eventual osteogenesis: for how long a graft can be left without being damaged, the optimal environment for the graft, and the deleterious effects of antibiotics on the graft and graft bed. Gray and Elves tried to answer these questions in a rat study [19]. The authors could demonstrate that the uptake of the used antibiotics had significant differences depending on the medium used as well as the storage period. Moreover, the use of any of the antibiotic preparations led to a highly significant drop in the relative index of osteogenesis compared with that of untreated controls. Similar findings could be shown in the histological examination of these grafts, indicating a decrease in the osteogenesis rate; however, this effect was antibiotic dose-dependent. On the contrary, Petri could not determine any statistical difference of the osteogenic activity between antibiotic-supplemented bone grafts and their controls in a pig model [29]. In this study, demineralized bone had a higher antibiotic uptake than the mineralized allografts in both the control the antibiotic-loaded group. Furthermore, demineralized grafts stimulated more bone growth than the mineralized allografts in the antibiotic-loaded group.

In a dog model, Lindsey and colleagues could not observe any histological, microradiographical, or biomechanical statistical differences after implantation of tobramycin-loaded bone grafts compared with a control group [25]. Serum tobramycin levels at 24 and 48 h were found to be below the normal therapeutical levels. Similar to that, Buttaro et al. investigated in a pig study the radiographical, histopathological, and immunohistochemical findings after incorporation of vancomycin-impregnated bone allografts [10]. Compared with a control group where bone allografts without antibiotics have been used, the authors could not determine any statistical differences between both groups regarding the radiographical bone healing, the rate of graft incorporation, or other similar histopathological or immunohistochemical parameters. Antibiotic concentrations could reach up to 220 times the MIC (minimum inhibitory concentration) at a ratio of 1 g vancomycin/300 g bone allografts. On the contrary, tetracycline deposited locally was shown by Gudmundson to markedly inhibit bone graft incorporation [44].

## 7. Clinical Experience

In clinical practise, AIBGs might be used in the prophylaxis as well as in the treatment of bone and joint infections (Table 2). The prophylactic use of antibiotic-loaded bone grafts for prevention of wound infections after orthopaedic surgery has been only sparsely discussed in the literature. To our knowledge, there exist only two studies which tried to investigate this prophylactic effect on the postoperative infection rate. Borkhuu and colleagues reported a significant decrease in the infection rate after spinal surgery in children with scoliosis because of cerebral palsy after local use of AIBGs compared with a group where plain bone grafts were inserted (from 15% to 4%) [9]. Khoo et al. determined the infection rate in 31 cases after insertion of 34 iontophoresed segmental allografts at the site of various orthopaedic surgeries in limb salvage [24]. There were no early infections, whereas two late infections were presumed to be hematogenous at a mean followup of 51 (24–82) months. 28 of the 34 allografts were retained.

Regarding the therapeutic use of AIBGs, some very encouraging results have been recently published about vancomycin-loaded bone grafts in the treatment of late infections after total hip replacement. In their latest report, Winkler et al. found an infection eradication rate of 92% in 37 patients at a mean followup of 4 years after one-stage uncemented revision [36]. After a two-stage revision in 29 cases, Buttaro et al. could observe a rate of recurrent infection of 3.3% at a mean followup of 32 months [11]. The success of their treatment led them to make the proposal that vancomycin-impregnated bone might also be used as antibiotic prophylaxis in patients undergoing revision THA for aseptic loosening. Similar favourable results have also been reported by English and colleagues after the use of vancomycin-, gentamicin-, or flucloxacillin-loaded bone grafts [18].

In another work by Buttaro et al. [12], the authors had the opportunity to histologically evaluate the behaviour of these allografts in one femoral and acetabular reconstruction. Both patients suffered from periprosthetic fractures that had to be revised. Low-magnification microscopy showed three areas on both biopsy specimens. Histologically normal laminar bone and periosteum were observed in the external area. Viable trabecular bone with polymethylmethacrylate was observed in the middle area of the specimen, as well as occasional macrophages, presumably in response to cement particles. Small islands of necrotic bone were observed in this zone. A variable amount of fibrotic tissue surrounding this bone was observed, as well as lymphocytes and plasma cells. The internal area showed fragments of necrotic and viable bone, interpreted as incorporated bone graft. The small fragments of necrotic trabecular bone were gradually replaced by viable new bone, described as creeping substitution, suggesting that the necrotic bone was allograft. Acute inflammation or wear debris was not evident. Based on these findings, the authors concluded that high levels of vancomycin do not affect incorporation of bone graft in a clinical setting. 

Besides their promising use in the treatment of infections orthopaedic surgery, equally successful results have been published from trauma surgery. Chan and colleagues evaluated in their latest report 96 patients treated for infected tibial non-unions [15]. All patients were managed with local antibiotic bead therapy and staged antibiotic-impregnated (*n* = 46) or pure autogenous cancellous bone graft (*n* = 50). In the first group, the infection eradication rate was 95% at an average followup of 4.8 years, whereas in the second group the rate was 82% at an average followup of 4.5 years (significant difference). The bony union rate was 100% in the first and 98% in the second group, respectively. In a similar report, Chen et al. [16] could observe a 100% infection eradication rate in 18 patients suffering from small infected tibial defects at an average followup of 48 months after vancomycin-impregnating bone grafting [16]. Bone union was achieved in 72% of the cases at an average of 5.8 months. The authors attributed the inferior bone union rate compared with the results of Chan et al. [15] to the lower vancomycin dose used. 

There exists a single study which reports on the clinical use of xenografts in the treatment of chronic osteomyelitis. Seber et al. presented seven cases that could be successfully treated by use of gentamicin-loaded xenografts at an average followup of 3.5 years [33]. The authors stated that although the osteogenic potential of xenografts is inferior to that of autologous bone, in some cases it is impossible to fill the large cavities with autografts, and morbidity from the donor site may follow.

In some cases with massive bone loss due to chronic infection, the surgeon has often limited reconstructive possibilities. The use of structural allografts might be a possible solution since these allografts allow the retainment of soft tissues and conventional revision prostheses may still be used. On the other side, concerns may be raised because the allograft is certainly avascular during the early postoperative period and carries a high risk of bacterial colonization, and, hence, emergence of clinical infection. However, Michalak and colleagues could demonstrate that the use of iontophoresed segmental allografts might be an effective option in the management of infections in revision arthroplasty [27]. Twelve patients undergoing two-stage revision for infection with major uncontained bony defects were treated by implantation of structural allografts which were loaded with gentamicin and flucloxacillin via iontophoresis. At a mean followup of 47 months none of the patients have become reinfected and all allografts remained in situ, giving an allograft retention rate of 100%. Only in one case, a further bone grafting had to be performed due to non-union 26 months after the primary surgery. 

## 8. Bacterial Adherence

Although autologous bone is the gold standard for bone restoration, donor site morbidity and limited bone volume have led to increased utilization of allograft bone. Approximately, 800,000 bone allograft transplantations are performed yearly in the United States [23]. Despite extended antibiotic prophylaxis, the reported incidence of graft colonization remains at 4% to 12% [23]. Like metallic implants, allografts act as highly porous, noncellular, and avascular foreign bodies that are prone to bacterial adhesion. Once bacteria are attached, they secrete a thick glycocalyx matrix rendering them inaccessible to immune surveillance and local cellular defense mechanisms [23]. Additionally to this problem, bacterial colonization might also occur in AIBGs when the antibiotics are eluted in insufficient amounts (below the MIC) and bacteria have survived despite the initial high-antibiotic concentrations. Moreover, the type as well as the site of donor site might also lead to different contamination rates. 

Ketonis and colleagues tried to assess the in vitro antimicrobial properties of allograft bone against a *S. aureus* strain [23]. Three groups were tested in this study: bone without antibiotics, bone where vancomycin was added as solution 12 hours after the initial bacterial contamination, and bone loaded with vancomycin from the beginning. Vancomycin-impregnated allografts demonstrated significantly better antimicrobial properties compared with the other groups. The authors could observe that *S. aureus* was capable of attaching and initiating biofilm formation within 6 hours. It appeared that bacterial colonization was potentiated by the porous nature of bone that provides bacteria with topographically protected niches where they can attach, proliferate, and form a mature biofilm.

## 9. Systemic Toxicity

Ideally, local antibiotic carriers should release high-antibiotic concentrations that vastly exceed those after systemic administration with low or no systemic toxicity. It is known that although other antibiotic-loaded devices such as acrylic bone cement are regarded to be safe; a remaining risk still exists [45].

Regarding the systemic toxicity of AIBGs, literature data are scarce. In 1986, McLaren and Miniaci presented an in vivo study that explored the effectiveness of antibiotic delivery by using morselized cancellous bone graft as a vehicle for powdered tobramycin [5]. They concluded that local treatment with antibiotic beads could be achieved for up to 3 weeks without toxic serum concentrations. In 1988, McLaren again found low concentrations of antibiotic in serum and urine, despite high local tissue levels [6]. This finding prompted him to recommend tobramycin-impregnated cancellous bone graft as an effective method of acutely reconstructing bone loss in compound fractures. 

Witsø et al. studied the systemic concentrations after local insertion of netilmicin- and vancomycin-loaded bone grafts in a rabbit model [39]. In animals operated on with implantation of netilimicin-loaded bone, mean peak concentrations were 4.2 (3.7-4.7) *μ*g/mL 2-3 h postoperatively, whereas vancomycin could not be detected at all in serum. Winkler et al. could measure a mean postoperative serum level of vancomycin of 0.2 (0.0–1.8) *μ*g/mL on the first postoperative day [36]. In 37 cases the renal function did not show any remarkable changes postoperatively. Seber et al. determined the gentamicin urine levels after insertion of gentamicin-loaded xenografts in the treatment of osteomyelitis [33]. Mean levels at 24 h were 4 *μ*g/mL, falling below the effective level of 0.5 *μ*g/mL after 8 days. No cases of nephrotoxicity or ototoxicity could be observed.

## Figures and Tables

**Table 1:** Literature review about antibiotic-impregnated bone grafts with specific attention on types of bone graft, antibiotics, and impregnation details.

Study	Publication year	In vitro/animal/in vivo	Bone graft	Antibiotic(s)	Antibiotic/bone graft ratio	Impregnation method	Duration of antibiotic impregnation
Allende et al. [8]	2010	In vivo	Cancellous/ corticocancellous	Vancomycin	1.5 g/case	n.r.	n.r.
Borkhuu et al. [9]	2008	In vivo	Corticocancellous	Gentamicin	8–10 mg/kg patient/30–60 cc	Solution	n.r.
Buttaro et al. [10]	2003	Animal	Cancellous	Vancomycin	20 mg/6 g	Manual mixing	15 min
Buttaro et al. [11]	2005	In vivo	Cancellous	Vancomycin	1 g/femoral head	Manual mixing	15 min
Buttaro et al. [12]	2005	In vivo	Cancellous	Vancomycin	0.5 g/femoral head	Manual mixing	15 min
Buttaro et al. [13]	2005	In vivo	Cancellous	Vancomycin	1 g/femoral head	Manual mixing	15 min
Chan et al. [14]	1998	In vivo	Cancellous	Vancomycin piperacillin	n.r.	Manual mixing	n.r.
Chan et al. [15]	2000	In vivo	Cancellous	Vancomycin piperacillin	n.r.	Manual mixing	n.r.
Chen et al. [16]	2005	In vivo	Cancellous	Vancomycin	0.5 g/16 g	Manual mixing	n.r.
Day et al. [17]	2005	In vitro	Cortical	Gentamicin flucloxacillin	1% solution of each antibiotic/graft	Iontophoresis	1/5/10 min
English et al. [18]	2002	In vivo	Cancellous	Gentamicin vancomycin flucloxacillin	n.r.	n.r.	n.r.
Gray and Elves [19]	1981	Animal	Corticocancellous	Benzylpenicillin streptomycin	800,000 units/L/n.r.	Solution	n.r.
					500 mg/L/n.r.		
Kanellakopoulou et al. [20]	2008	In vitro	Cancellous	Fusidic acid teicoplanin	100 mg/mL	Solution	60 min
Kanellakopoulou et al. [21]	2010	In vitro	Cancellous	Moxifloxacin	500 mg/1 g	Solution	n.r.
Ketonis et al. [22]	2010	In vitro	Corticocancellous	Vancomycin	10 mg/mL	Solution	12–16 h
Ketonis et al. [23]	2010	In vitro	Cortical morselized	Vancomycin	10 mg/mL	Solution	n.r.
Khoo et al. [24]	2006	In vivo	Segmental allograft	Gentamicin flucloxacillin	1% gentamicin solution/graft 4% flucloxacillin solution/graft	Iontophoresis	20 min
Lindsey et al. [25]	1993	Animal	Cancellous	Tobramycin	90 mg/3 g	Manual mixing	n.r.
					0.5 *μ*g/10 *μ*L		
					2.5 *μ*g/10 *μ*L		
				Cefazolin	10 *μ*g/10 *μ*L		
					60 *μ*g/10 *μ*L		
Mathijssen et al. [26]	2010	In vitro	Cancellous		100 *μ*g/10 *μ*L	Solution	n.r.
					0.5 *μ*g/10 *μ*L		
					2.5 *μ*g/10 *μ*L		
				Vancomycin	10 *μ*g/10 *μ*L		
					70 *μ*g/10 *μ*L		
					100 *μ*g/10 *μ*L		
McLaren [7]	1989	In vivo	Cancellous	Vancomycin tobramycin	1 g/>20 g 720 mg/>20 g	Manual mixing	n.r.
Michalak et al. [27]	2006	In vivo	Segmental allograft	Gentamicin flucloxacillin	1% gentamicin solution/graft 4% flucloxacillin solution/graft	Iontophoresis	20 min
Miclau et al. [28]	1993	In vitro	Cancellous	Tobramycin	25 mg/g	Manual mixing	n.r.
Petri [29]	1984	Animal	Bone-gelatin powder	Cephalothin tobramycin	100–200 *μ*g/mL b-g powder 1-2 mg/mL b-g-powder	Manual mixing	n.r.
Petri and Schaberg [30]	1984	Animal	Bone-gelatin powder	Cephalothin tobramycin	200 *μ*g/mL b-g powder 2 mg/mL b-g powder	Manual mixing	n.r.
Petri [31]	1991	Animal	Bone-gelatin powder	Cephalothin tobramycin	1 mg/mL b-g powder 1 mg/mL b-g powder	Manual mixing	n.r.
Rhyu et al. [32]	2003	In vitro	Cancellous	Vancomycin	125 mg/mL/66–70 mg	Solution	24 h
Seber et al. [33]	1998	In vivo	Xenograft	Gentamicin	25 mg/125 cc	Solution	8 h
Winkler et al. [34]	2000	In vitro	Cancellous cortical	Vancomycin tobramycin	100 mg/mL/block 80 mg/mL/block	Solution	24 h
Winkler et al. [35]	2006	In vivo	Cancellous	Vancomycin tobramycin	100 mg/cc 75 mg/cc	Solution	24 h
Winkler et al. [36]	2008	In vivo	Cancellous	Vancomycin tobramycin	100 mg/cc 75 mg/cc	Solution	24 h
Witsø et al. [37]	1999	In vitro	Cancellous	Benzylpenicillin dicloxacillin cephalothin netilmicin vancomycin clindamycin ciprofloxacin rifampicin	100 mg/mL/g 100 mg/mL/g 100 mg/mL/g 100 mg/mL/g 50 mg/mL/g 150 mg/mL/g 2 mg/mL/g 60 mg/mL/g	Solution	10 min
Witsø et al. [38]	2000	In vitro/animal	Cancellous	Benzylpenicillin cephalothin clindamycin netilmicin vancomycin ciprofloxacin rifampicin	100 mg/mL/g 100 mg/mL/g 150 mg/mL/g 100 mg/mL/g 50 mg/mL/g 2 mg/mL/g 60 mg/mL/g	Solution	10 min
Witsø et al. [39]	2002	In vitro/animal	Cancellous	Netilmicin vancomycin	100 mg/mL/g 100 mg/mL/g	Solution	10 min
Witsø et al. [40]	2004	In vivo	Cancellous	Netilmicin	50/100 mg/mL/50 g	Solution	10 min
Witsø et al. [41]	2005	In vitro	Cortical	Netilmicin vancomycin ciprofloxacin rifampicin	400 mg/mL/1.46 g 100 mg/mL/1.46 g 2 mg/mL/1.46 g 50 mg/mL/1.46 g	Solution	1/10/100 h

n.r.: not reported.

**Table 2:** Clinical experience with use of antibiotic-loaded bone grafts in orthopedic and trauma surgery.

Study	Number of patients (*n =*)	Surgical indication	Infection eradication	Surgery- and infection-related complications	Followup (months)
Allende et al. [8]	12	Posttraumatic infected nonunion of humerus, ulna, and radius	100%	1x transitory radial nerve neurapraxia	19 [12–96]
Borkhuu et al. [9]	154	Spinal fusion for scoliosis	96%	none	31 [11–52]
Buttaro et al. [11]	29	THA	97%	1x periprosthetic fracture 2x prosthesis dislocations 4x displacement of greater trochanter	32 [24–60]
Chan et al. [14]	36	Infected tibial nonunions	94%	2x pin tract infection 2x allergic reactions to antibiotics	44 [36–60]
Chan et al. [15]	46	Infected tibial nonunions	96%	3x pin tract infection 2x allergic reactions to antibiotics	58 [48–72]
Chen et al. [16]	18	Infected tibial nonunions	100%	5x pin tract infection 1x knee stiffness	48 [24–74]
English et al. [18]	^ ∗^	THA	^ ∗^	^ ∗^	^ ∗^
Khoo et al. [24]	31	THA, TKA, tumor surgery, and hip joint fracture	90%	4x non-union 3x fracture 1x ischemia requiring amputation	55 [24–89]
McLaren [7]	17	n.r.	93%	n.r.	n.r.
Michalak et al. [27]	12	THA, TKA		2x hip-, 1x knee dislocation 1x non-union	47 [14–78]
Seber et al. [33]	7	Postoperative and traumatic chronic OM	100%	none	42 [42–60]
Winkler et al. [35]	48	37x THA 6x TKA 3x failed intramedullary nailing 2x knee empyema	96%	none	38 [12–84]
Winkler et al. [36]	37	THA	92%	none	53 [24–96]

THA: total hip arthroplasty; TKA: total knee arthroplasty; n.r.: not reported; OM: osteomyelitis.

^∗^Unclear study data; only some of the patients have been treated with AIBGs.
